# Temporal Expression of NLRP3 Inflammasome Components in Patients with Acute Coronary Syndrome

**DOI:** 10.3390/life16010001

**Published:** 2025-12-19

**Authors:** Paraskevi Papanikolaou, Andreas Aggelopoulos, Alexios S. Antonopoulos, Panagiotis Theofilis, Maria Gazouli, Konstantinos Tsioufis, Dimitris Tousoulis

**Affiliations:** 1 1st Cardiology Department, Hippokration Hospital, National Kapodistrian University of Athens, 11527 Athens, Greece; evipapanikolaou215@gmail.com (P.P.);; 2 Department of Biology, School of Medicine, National and Kapodistrian University of Athens, 11527 Athens, Greece

**Keywords:** inflammation, NLRP3 inflammasome, acute coronary syndrome

## Abstract

**Background:** Inflammation is a central driver of atherothrombosis, yet the temporal behavior of key inflammasome mediators following acute coronary syndrome (ACS) is not well characterized. The NLRP3 inflammasome, a major regulator of interleukin (IL)-1β activation, has been implicated in plaque destabilization and recurrent cardiovascular risk. This study aims to investigate the temporal expression of NLRP3 inflammasome components in peripheral blood mononuclear cells (PBMCs) of patients with ACS. **Methods:** In this prospective observational study, PBMCs were collected from 73 patients with ACS during the early in-hospital phase and at 8–12 weeks follow-up. Gene expression of NLRP3, caspase-1, and IL-1β was quantified by qRT-PCR, and fold-change was calculated using the 2^−ΔΔCT^ method. Associations with clinical and biochemical variables were evaluated using multivariable linear regression. **Results:** Expression of all measured inflammasome-related genes increased significantly at follow-up compared with baseline: caspase-1 (≈2-fold, *p* = 0.003), NLRP3 (>10-fold, *p* < 0.001), and IL-1β (≈4-fold, *p* < 0.001). Subgroup analyses showed that the post-ACS upregulation of NLRP3, caspase-1, and IL-1β was consistent across STEMI and NSTEMI presentations and was not significantly modified by diabetes status. Caspase-1 fold-change correlated positively with IL-1β, LDL-cholesterol, peak troponin I, and high sensitivity C reactive protein, whereas NLRP3 showed minimal correlations with clinical variables. In multivariable analysis, caspase-1 upregulation was independently associated with STEMI presentation and low-density lipoprotein-cholesterol, and IL-1β with type 2 diabetes. **Conclusions:** Patients with ACS exhibit significant and persistent upregulation of NLRP3 inflammasome components weeks after the acute event, indicating sustained immune cell priming during recovery. These findings highlight a potential molecular substrate for residual inflammatory risk and support further exploration of inflammasome-targeted therapies in the post-ACS period.

## 1. Introduction

Acute coronary syndromes (ACSs) remain a leading cause of morbidity and mortality worldwide, arising from a complex interplay between atherosclerotic plaque instability, thrombosis, and inflammation [1]. Beyond lipid accumulation and plaque rupture, inflammation plays a decisive role in the initiation, progression, and clinical expression of atherosclerosis [2]. A growing body of evidence supports the notion that immune activation persists even after the acute ischemic event, contributing to adverse remodeling and recurrent cardiovascular events [3].

Among the inflammatory mediators implicated in atherothrombosis, the NLRP3 inflammasome has emerged as a pivotal component of innate immunity [4]. NLRP3 acts as a cytosolic sensor that assembles with the adaptor ASC and caspase-1 to form a multiprotein complex responsible for activating caspase-1, which then converts pro–interleukin (IL)-1β and pro–IL-18 into their mature, biologically active forms [5]. These cytokines amplify the inflammatory response, promoting leukocyte recruitment, endothelial dysfunction, and tissue injury [5]. Activation of the NLRP3 inflammasome can be triggered by cholesterol crystals, oxidized lipoproteins, and reactive oxygen species [6]—all relevant to the atherosclerotic milieu.

Experimental and clinical studies have shown that excessive inflammasome activation contributes to plaque progression and rupture [7,8,9]. Inhibition or genetic deletion of NLRP3 or IL-1β attenuates lesion development and improves plaque stability in preclinical models [9,10]. Furthermore, in the CANTOS trial, selective IL-1β blockade reduced recurrent myocardial infarction independently of lipid lowering [11], confirming the clinical importance of inflammasome signaling in residual inflammatory risk.

Despite these insights, the temporal expression of NLRP3 inflammasome components during and after ACS remains unclear. While the early in-hospital phase is characterized by robust inflammation, whether inflammasome activation persists during recovery has not been systematically examined. The present study, therefore, aimed to characterize the temporal expression patterns of NLRP3, caspase-1, and IL-1β in peripheral blood mononuclear cells (PBMCs) from patients with ACS, both in the early in-hospital phase and at follow-up.

## 2. Methods

### 2.1. Study Design

This was a prospective, observational study conducted at the 1st Cardiology Department of Hippokration General Hospital, Athens, Greece, between June 2019 and September 2022. Consecutive adult patients admitted with a diagnosis of ACS were screened for eligibility and enrolled after providing written informed consent. The study was conducted in accordance with the Declaration of Helsinki and approved by the institutional ethics committee (protocol code: 49300, date of approval: 13 May 2019).

Adult patients (≥18 years) admitted with a diagnosis of ACS, including ST-segment elevation myocardial infarction (STEMI) or non–ST-segment elevation myocardial infarction (NSTEMI), were screened for eligibility. ACS diagnosis was established according to current European Society of Cardiology (ESC) guidelines, based on clinical presentation, electrocardiographic findings, and cardiac biomarker elevation [12].

Patients were excluded if any of the following criteria were present at screening:

- active infection
- autoimmune disease
- chronic inflammatory disease
- known malignancy
- current treatment with systemic corticosteroids or other immunosuppressive agents
- unwillingness or inability to provide written informed consent.

All patients were treated according to contemporary ESC guidelines for the management of ACS [12]. Reperfusion therapy for STEMI was performed with primary PCI as the default strategy, and NSTEMI patients underwent early invasive evaluation (within 24 h) with PCI [12]. Optimal medical therapy was administered to all patients during hospitalization and at discharge, including antithrombotic therapy (dual antiplatelet therapy or single antiplatelet therapy and anticoagulation in the presence of anticoagulation indication), high intensity statin therapy, beta blockers, and renin-angiotensin system blockers (RASb), unless contraindicated [12].

Clinical and demographic characteristics, cardiovascular risk factors, and pre-admission use of aspirin, P2Y12 inhibitor, statin, beta blocker, and RASb were recorded for all participants. Peripheral blood samples were collected from each patient at two time points: early in-hospital phase (baseline): within 24 h of hospital admission; Follow-up phase: at 8–12 weeks post-discharge during an outpatient visit. Serum total cholesterol, low-density lipoprotein cholesterol (LDL-C), and high-density lipoprotein cholesterol (HDL-C) levels were measured at baseline.

### 2.2. NLRP3 Inflammasome Components Measurement

Total RNA extraction from all specimens was conducted using NucleoZOL (Macherey-Nagel, Düren, Germany). cDNA synthesis from the total RNA was accomplished utilizing the TAKARA kit (Takara Bio Europe SAS, Saint-Germain-en-Laye, France) according to the manufacturer’s instructions. Expression levels of IL1b, NLRP3 and Casp1 were measured via quantitative real-time polymerase chain reaction (qRT-PCR). To perform qRT-PCR, the KAPA SYBR FAST qPCR mix (KAPA BIOSYSTEMS, Cape Town, South Africa) was employed. GAPDH was used as a reference gene. Duplicate reactions were conducted for all samples to guarantee reproducibility and gene expression was normalized to the expression of the relevant reference genes. The sequences of the primers used are shown in Table 1. Expression values were normalized to housekeeping genes, and relative expression was calculated using the comparative threshold cycle (ΔCT) method. Fold change (follow-up vs. baseline) for each subject was then calculated by the 2^−ΔΔCT^ method, with the corresponding standard error of mean.

### 2.3. Statistical Analysis

Continuous variables were tested for normality of distribution with the Kolmogorov–Smirnov test, and are presented as mean ± standard deviation or median (interquartile range). Categorical variables are displayed as frequencies and percentages. A Student’s *t*-test was used to compare differences between two groups of normally distributed continuous data. Differences between categorical variables were tested by forming contingency tables and performing χ^2^-tests. Analysis of variance (ANOVA) for repeated measures was used to assess the over-time changes in NLRP3-related mediators. A sensitivity analysis with the follow-up duration as a covariate was also performed, as well as interactions according to ACS type and DM status. Multivariable linear regression was performed to determine predictors of NLRP3 components fold change at follow-up. We also conducted a correlation analysis using the was assessed using the Spearman correlation coefficient. A two-sided *p*-value < 0.05 was considered statistically significant. Analyses were performed using SPSS version 28.0 (IBM Corp, Armonk, NY, USA).

## 3. Results

### 3.1. Baseline Characteristics of the Study Population

Among patients with ACS screened for eligibility, 84 patients were enrolled (Figure S1). After exclusion of 11 patients who were lost to follow-up, 73 consecutive patients with ACS with completed paired PBMC samples were analyzed (NSTEMI: 39, STEMI: 34). Their mean age was 71 years and 71% were male, with a high prevalence of cardiovascular risk factors (hypertension: 78.1%, type 2 diabetes mellitus (T2DM): 45.2%, dyslipidemia: 68.5%, smoking: 54.8%) (Table 2). Prior to admission, 41.1% of patients were receiving aspirin, 17.8% a P2Y12 inhibitor, 57.5% a statin, 63.0% a RASb, and 47.9% a beta blocker. Baseline lipid measurements revealed elevated total cholesterol and LDL-C levels, with relatively low HDL-C.

When comparing patients with STEMI and NSTEMI, there were no differences in baseline characteristics apart from a significantly higher prevalence of dyslipidemia in patients with NSTEMI (NSTEMI: 84.6% vs. STEMI: 50%, *p* = 0.001) (Table 3). Pre-admission statin use was more frequent in NSTEMI than STEMI (71.8% vs. 41.2%, *p* = 0.008), whereas pre-admission use of aspirin, P2Y12 inhibitors, RASb, and beta blockers did not differ significantly between groups. Despite a higher prevalence of dyslipidemia and greater pre-admission statin use in NSTEMI patients, baseline total cholesterol, LDL-C, and HDL-C levels did not differ significantly between NSTEMI and STEMI presentations.

### 3.2. Expression of NLRP3 Inflammasome Components

**Figure 1:**
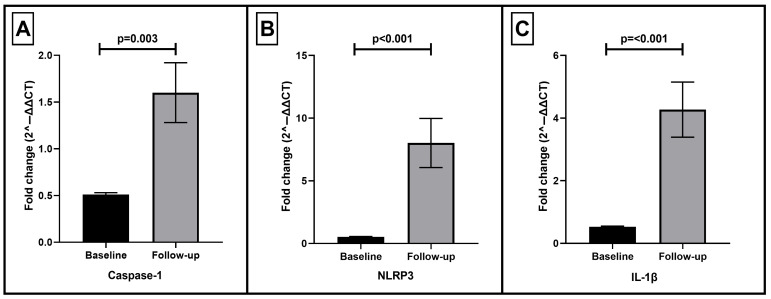
Longitudinal expression of NLRP3 inflammasome components in patients after acute coronary syndrome. Relative mRNA expression levels of key inflammasome components were assessed at baseline and during follow-up using quantitative RT-PCR. Bars represent mean ± standard error of the mean (SEM) of fold change values calculated as 2^−ΔΔCT^. (**A**) Caspase-1 expression significantly increased at follow-up compared with baseline (*p* = 0.003). (**B**) NLRP3 expression showed a marked upregulation (*p* < 0.001). (**C**) IL-1β expression was also significantly elevated (*p* < 0.001). These findings indicate activation of the NLRP3 inflammasome pathway during the post-ACS period. Expression of NLRP3 inflammasome components in peripheral blood mononuclear cells at baseline and follow-up after acute coronary syndrome. Bars show the arithmetic mean fold change ± SEM for (**A**) caspase-1, (**B**) NLRP3, and (**C**) IL-1β. Statistical comparisons were performed using two-sided paired *t*-tests.

The median follow-up duration was 9 months (IQR 9–10 months). The expression of inflammatory mediators did not differ at baseline with regard to ACS type. As shown in Figure 1, the expression of major NLRP3 inflammasome pathway components significantly increased during follow-up compared to baseline in patients with acute coronary syndrome. Specifically, Caspase-1 expression (panel A) demonstrated a nearly 2-fold increase at follow-up relative to baseline (*p* = 0.003). NLRP3 expression (panel B) showed an even greater upregulation, rising more than 10-fold (*p* < 0.001), indicating marked activation of the inflammasome complex. Similarly, IL-1β expression (panel C) was significantly elevated at follow-up (approximately 4-fold increase, *p* < 0.001).

Sensitivity analysis adjusting for the exact follow-up interval demonstrated that follow-up duration did not significantly modify the temporal change in gene expression for NLRP3 (*p* = 0.29), caspase-1 (*p* = 0.18), and IL-1β (*p* = 0.77).

### 3.3. Expression of NLRP3 Inflammasome Components by ACS Type and T2DM Status

**Figure 2:**
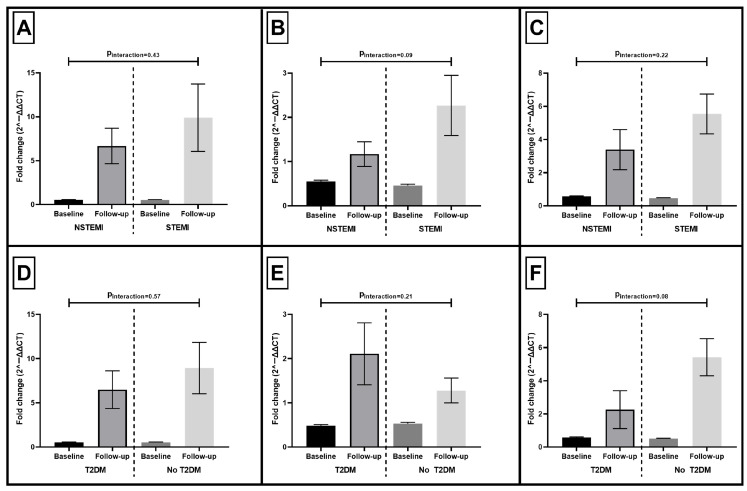
Temporal changes in NLRP3 inflammasome gene expression by ACS type and T2DM status. Fold-changes (2^−ΔΔCT^) in caspase-1 (Panels **A**,**D**), NLRP3 (Panels **B**,**E**), and IL-1β (Panels **C**,**F**) at baseline and follow-up are shown for (**A**–**C**) NSTEMI vs. STEMI and (**D**–**F**) patients with vs. without type 2 diabetes mellitus (T2DM). Bars represent mean ± SEM. All inflammasome components increased significantly from baseline to follow-up in each subgroup. P_interaction_ values indicate the significance of the time × subgroup interaction from repeated-measures ANOVA.

To explore whether temporal changes in inflammasome activation differed across clinical subgroups, we compared fold-changes in NLRP3, caspase-1, and IL-1β expression according to ACS type (NSTEMI vs. STEMI) and the presence of T2DM (T2DM vs. no T2DM). Across all three inflammasome components, expression increased significantly from baseline to follow-up in both NSTEMI and STEMI groups. However, the magnitude of upregulation did not differ significantly between ACS types (Figure 2). Notably, the temporal increase in inflammasome-related gene expression was similarly evident in both patients with and without T2DM.

### 3.4. Correlations of NLRP3 Inflammasome Components

**Figure 3:**
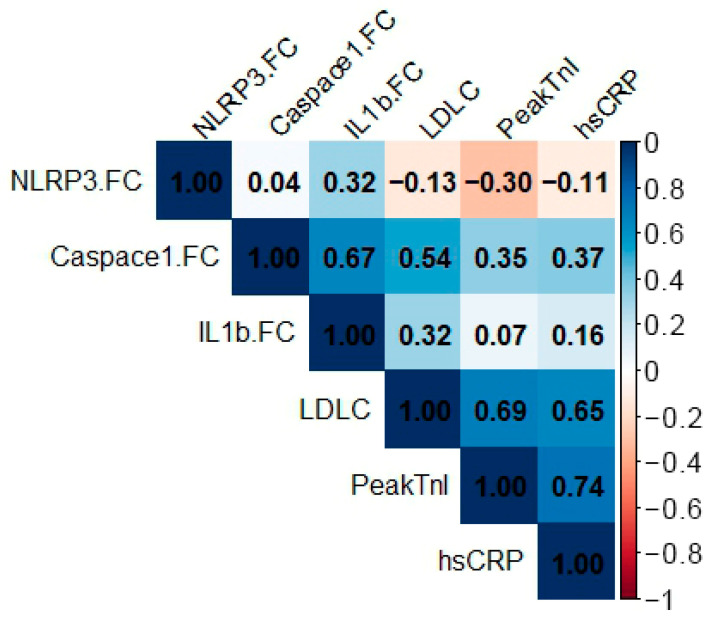
Correlation heatmap between inflammasome-related gene expression and biochemical markers. The matrix displays correlation coefficients between fold change in NLRP3 (NLRP3.FC), caspase-1 (Caspase1.FC) and interleukin-1β (IL1b.FC) and circulating low-density lipoprotein cholesterol (LDL-C), peak troponin I (PeakTnI) and high-sensitivity C-reactive protein (hsCRP). Numeric values in each cell represent correlation coefficients, and the color scale (from red to dark blue) indicates the direction and magnitude of the association (−1 to +1).

Correlation analysis is displayed in Figure 3. Caspase-1 fold change (FC) showed the strongest associations in the inflammasome axis, correlating positively with IL-1β FC (r = 0.67), LDL-C (r = 0.54), peak troponin I (r = 0.35), and hsCRP (r = 0.37). IL-1β FC correlated modestly with LDL-C (r = 0.32) and weakly with peak troponin I (r = 0.07) and hsCRP (r = 0.16). In contrast, NLRP3 FC showed minimal relationships with other variables (e.g., Caspase-1 FC r = 0.04; IL-1β FC r = 0.32; LDL-C r = −0.13; peak troponin I r = −0.30; hsCRP r = −0.11).

### 3.5. Predictors of NLRP3 Inflammasome Components Deregulation

Multivariable linear regression analyses were performed to identify independent determinants of inflammasome-related gene expression fold change at follow-up (Table 4). distinct associations were observed for downstream components of the inflammasome pathway. NLRP3 was largely independent of baseline demographic characteristics, cardiovascular risk factors, lipid parameters, infarct presentation, inflammatory markers, or pre-admission medication use. Caspase-1 fold change was independently associated with STEMI presentation (B = 3.50, 95% CI 1.00–6.00, *p* = 0.012) and baseline LDL-cholesterol levels (B = 0.08 per mg/dL, 95% CI 0.02–0.14, *p* = 0.01. For IL-1β, T2DM (B = −4.81, 95% CI −8.17 to −1.46, *p* = 0.02) was significantly associated with lower fold change, even though baseline IL-1β expression did not differ by T2DM status (T2DM: 0.51 ± 0.14 vs. no T2DM: 0.52 ± 0.11, *p* = 0.83). Baseline LDL-C was also independently associated with IL-1β upregulation (B = 0.33, 95% CI 0.08–0.22, *p* = 0.01). Among pre-admission medications, statin use was independently associated with higher IL-1β fold change (B = 8.08, 95% CI 1.67–14.49, *p* = 0.03), while no consistent associations were observed for aspirin, P2Y12 inhibitors, beta blockers, or RASb.

## 4. Discussion

In this study, we demonstrated a significant upregulation of NLRP3, IL-1β, and Caspase-1 gene expression in PBMCs of patients following ACS compared with the early in-hospital phase. These findings indicate persistent activation of the inflammasome pathway after the ischemic event, involving both its priming (NLRP3, IL-1β) and effector (Caspase-1) components. This sustained transcriptional response may reflect a prolonged inflammatory activation, potentially contributing to the residual inflammatory risk commonly observed in patients after myocardial infarction.

Inflammation plays a critical role in the pathogenesis of atherosclerosis and its clinical complications. The NLRP3 inflammasome serves as a molecular platform that senses metabolic and oxidative stress signals, linking lipid accumulation and cell injury to immune activation [4]. Upon activation, NLRP3 recruits the adaptor ASC and pro-caspase-1, leading to caspase-1 activation and subsequent cleavage of pro-IL-1β and pro-IL-18 into their mature forms [4]. These cytokines amplify local and systemic inflammation, promoting endothelial dysfunction, leukocyte adhesion, and matrix degradation within atherosclerotic plaques [4]. Consequently, NLRP3 activation contributes not only to plaque progression but also to plaque destabilization and thrombus formation. These molecular findings should also be interpreted in the broader context of systemic inflammatory markers commonly used in ACS.

Beyond molecular inflammasome activation, systemic inflammation after ACS is frequently assessed using composite leukocyte-derived indices, such as the neutrophil-to-lymphocyte ratio (NLR), systemic immune–inflammation index (SII), and systemic inflammation response index (SIRI) [13,14,15]. While they capture systemic inflammation at a cellular level, they do not directly interrogate intracellular inflammasome activation, which is associated with innate immune signaling. Since these indices were not the focus of the present study and were therefore not formally analyzed, future studies integrating leukocyte-derived inflammatory indices with molecular inflammasome profiling may help delineate the interplay between systemic inflammatory burden and inflammasome activation after ACS.

Our results align with previous observations indicating that NLRP3 and IL-1β expression are upregulated during ACS [16,17]. However, by assessing two distinct time points, our study adds a temporal dimension to this relationship. The significant increase in NLRP3 and IL-1β expression at the mid-term follow-up suggests that inflammasome priming persists beyond the acute event, indicating incomplete resolution of inflammation. Such sustained immune activation could be responsible for the residual inflammatory risk driving recurrent ischemic events.

The subgroup analyses further support the notion that inflammasome activation after ACS represents a generalized post-ischemic biological response rather than one confined to specific clinical phenotypes. The magnitude of upregulation in NLRP3, caspase-1, and IL-1β did not differ significantly between patients with STEMI and NSTEMI, indicating that the sustained transcriptional activation of the inflammasome pathway is not solely a function of infarct presentation or presumed ischemic burden. Similarly, T2DM did not significantly modify the temporal expression patterns of inflammasome components. The absence of significant interactions suggests that the transcriptional priming of PBMCs after ACS occurs across a broad spectrum of clinical backgrounds. These findings align with the concept of persistently enhanced immune activation independent of traditional modifiers such as ischemic phenotype or metabolic comorbidity.

Our multivariable analyses further clarify the determinants of post-ACS inflammasome activation. While NLRP3 upregulation appeared independent of traditional risk factors, infarct size, or systemic inflammation, selective associations emerged for downstream components of the pathway. Caspase-1 activation was linked to STEMI presentation and LDL-cholesterol levels, whereas IL-1β showed an independent relationship with LDL. These patterns suggest that although inflammasome priming after ACS is widespread, its effector activation may be modulated by specific metabolic and clinical factors.

An unexpected finding was the independent association between T2DM and a lower IL-1β fold-change. Although T2DM is frequently linked to heightened inflammasome activity [18], baseline IL-1β expression was similar between participants with and without T2DM, suggesting that the observed association reflects a relatively attenuated post-ACS transcriptional upregulation rather than a higher starting point. Several mechanisms may account for this pattern. Antidiabetic therapies with anti-inflammatory properties may prevent IL-1β/NLRP3 pathway activation [19,20], while chronic metabolic inflammation and subsequent trained immunity [21] may also lead to altered innate immune responsiveness following an acute ischemic event. Because detailed post-discharge antidiabetic therapy and glycemic control were not captured, these explanations remain speculative and should be evaluated in future, adequately powered cohorts.

The underlying mechanisms responsible for sustained NLRP3 expression remain speculative but may involve several pathways. Persistent metabolic stress, such as hyperglycemia or dyslipidemia, can maintain inflammasome priming via reactive oxygen species and mitochondrial dysfunction [18]. In addition, monocyte subsets mobilized after myocardial infarction, particularly the pro-inflammatory CD14++CD16− population [22], may contribute disproportionately to NLRP3 upregulation. Epigenetic reprogramming of immune cells, often termed trained immunity [23], may also play a role by conferring long-lasting pro-inflammatory potential following an initial insult. This concept provides a plausible explanation for the enduring nature of inflammasome activation observed in our cohort.

Previous work has highlighted the importance of persistent inflammation after myocardial infarction. A prior study has shown that the most widely available inflammatory marker, hsCRP, remains elevated for months and predicts recurrent events [24]. Klingenberg et al. demonstrated that residual inflammatory risk at 12 months after ACS was common and associated with adverse outcomes [3]. Our findings complement these data by identifying a molecular signature of inflammasome priming that may contribute to this residual risk.

Experimental studies provide further support. NLRP3 knockout mice exhibit reduced infarct size and improved ventricular remodeling following myocardial ischemia–reperfusion injury [25], while pharmacological inhibition of caspase-1 ameliorates vascular inflammation [26]. In humans, the CANTOS trial established IL-1β inhibition as an effective anti-inflammatory strategy that reduces recurrent myocardial infarction independent of lipid lowering [11]. Similarly, low-dose colchicine, an indirect NLRP3 inhibitor, has shown cardiovascular benefit in large clinical trials (COLCOT, LoDoCo2) [27,28]. These observations emphasize the clinical relevance of inflammasome activity in both early in-hospital and chronic phases of coronary disease.

However, few studies have examined the longitudinal expression of NLRP3 pathway genes in circulating immune cells. Our results extend prior cross-sectional findings by demonstrating a clear temporal upregulation of caspase-1, NLRP3, and IL-1β, supporting the idea that immune cells remain transcriptionally activated long after the ischemic insult. This may reflect sustained exposure to pro-inflammatory mediators, persistent oxidative stress, or ongoing recruitment of monocytes into the circulation.

The persistence of inflammasome activation has potential implications for risk stratification and therapy. Measurement of NLRP3-related gene expression in PBMCs could serve as a biomarker of post-ACS immune status, identifying patients at risk of prolonged inflammation and recurrent events. Furthermore, it may guide personalized anti-inflammatory therapy, enabling clinicians to target those most likely to benefit from interventions such as IL-1 blockade or colchicine. Our findings support the rationale for timing anti-inflammatory treatment beyond the acute hospitalization period. Most clinical trials have focused on early administration of anti-inflammatory agents, but persistent upregulation of inflammasome genes weeks after ACS suggests that later intervention might still be effective.

Several limitations should be acknowledged. First, the sample size was modest, and results should be interpreted as exploratory. This study was not formally powered to detect specific effect sizes in gene expression outcomes. The sample size reflects the number of eligible patients with paired PBMC samples at both time points, and the analyses should therefore be interpreted as hypothesis-generating. While the cohort is adequate for detecting moderate associations in multivariable models, smaller effects may not have been identified, and confidence intervals for several predictors remain wide. Larger, prospectively powered studies will be required to confirm these findings and more precisely characterize determinants of post-ACS inflammasome activation. Moreover, the absence of a non-ACS control group, such as patients with stable CAD or individuals without cardiovascular disease, makes it impossible to determine whether the follow-up expression levels of NLRP3, caspase-1, and IL-1β represent true post-ACS elevations in the setting of a stable atherosclerotic baseline, or whether they simply reflect the chronically elevated, low-grade inflammatory state known to characterize patients with established atherosclerosis. Future studies incorporating both stable CAD and non-CAD controls will be necessary to clearly delineate ACS-specific inflammasome activation patterns. Another limitation is the lack of detailed hemodynamic profiling at presentation, including Killip classification and the presence or absence of cardiogenic shock. These clinical parameters reflect the severity of acute myocardial dysfunction and could potentially influence the systemic inflammatory response, including inflammasome-related gene expression. Because these data were not systematically recorded in our cohort, we were unable to assess their contribution to the observed temporal changes. Additionally, only mRNA expression was measured, which may not fully reflect protein levels or enzymatic activity. Assessment of caspase-1 activity or circulating IL-1β concentrations would strengthen the biological interpretation. Third, baseline PBMC sampling was not performed on a fixed interval from symptom onset or reperfusion, and exact pre- versus post-PCI sampling times were not systematically captured. Thus, heterogeneity in the peri-procedural inflammatory milieu may have influenced baseline measurements. Importantly, although pre-admission use of major cardiovascular medications was recorded and all patients received contemporary guideline-directed medical therapy during hospitalization and at discharge, detailed post-discharge medication exposure at follow-up (including dose, intensity, adherence, medication changes, and additional drug classes with anti-inflammatory effects such as SGLT2 inhibitors, GLP-1 receptor agonists, or colchicine [29,30]) was not captured. Furthermore, lipoprotein(a) levels were not systematically measured and could not be evaluated, despite their recognized role in residual cardiovascular risk and inflammation [31]. Finally, although we observed a clear temporal pattern, a longer follow-up would be necessary to determine whether inflammasome activation eventually resolves or persists chronically.

Future studies should validate these findings in larger, multicenter cohorts and integrate protein-level and functional assays of inflammasome activity. Correlation with imaging markers of myocardial healing and clinical outcomes could clarify the prognostic value of persistent NLRP3 upregulation. Moreover, mechanistic studies exploring the triggers of sustained priming—such as metabolic alterations, immune cell phenotype shifts, or epigenetic modifications—will be critical to understanding how inflammation transitions from resolution to chronicity.

Translationally, it will be important to investigate whether targeted inhibition of NLRP3 or IL-1β after hospital discharge can modulate these transcriptional signatures and improve outcomes. The growing availability of specific NLRP3 inhibitors offers promising avenues for such personalized anti-inflammatory strategies.

## 5. Conclusions

In summary, our study reveals dynamic temporal changes in inflammasome gene expression following ACS, characterized by sustained upregulation of caspase-1, NLRP3 and IL-1β. These findings suggest persistent immune cell priming during recovery, potentially contributing to residual inflammatory risk. By elucidating this molecular pattern, we provide a foundation for future work on inflammasome-targeted diagnostics and therapies aimed at improving post-ACS outcomes.

## Figures and Tables

**Table 1:** Sequences of real-time PCR primers used in the study.

Name	Sequence (5′-3′)
Caspase-1	F: 5′ TGCCTGTTCCTGTGATGTGGAGGA 3′ R: 5′ CAGTGGTGGGCATCTGCGCT 3′
NLRP3	F: 5′-CACCTGTTGTGCAATCTGAAG-3′ R: 5′-GCAAGATCCTGACAACATGC-3′
IL-1β	F: CCACAGACCTTCCAGGAGAATG R: GTGCAGTTCAGTGATCGTACAGG
GAPDH	F: GTCTCCTCTGACTTCAACAGCG R: ACCACCCTGTTGCTGTAGCCAA

**Table 2:** Baseline characteristics of the study population.

Variable	
Age, years	71.0 ± 13.5
Male sex, %	71.2
BMI, kg/m^2^	29.2 ± 3.9
Hypertension, %	78.1
T2DM, %	45.2
Dyslipidemia, %	68.5
Family history of CAD, %	31.5
Current smoking, %	54.8
STEMI, %	46.6
NSTEMI, %	53.4
Prior medication use	
Aspirin, %	41.1
P2Y12 inhibitor, %	17.8
Statin, %	57.5
RASb, %	63
Beta blocker, %	47.9
Lipid profile	
Total cholesterol, mg/dL	209 (48)
LDL-C, mg/dL	125 (36)
HDL-C, mg/dL	33 (8)

BMI: body mass index, T2DM: type 2 diabetes mellitus, STEMI: ST elevation myocardial infarction, NSTEMI: non-ST elevation myocardial infarction, RASb: renin-angiotensin system blocker, LDL-C: low-density lipoprotein cholesterol, HDL-C: high-density lipoprotein cholesterol. Continuous variables are presented as mean ± standard deviation. Categorical variables are presented as percentages.

**Table 3:** Baseline characteristics of the study population according to ACS type.

Variable	NSTEMI	STEMI	*p*
Age, years	73.9 ± 12.0	68.7 ± 14.8	0.10
Male sex, %	71.8	70.6	0.91
BMI, kg/m^2^	30.3 ± 4.2	29.0 ± 3.5	0.14
Hypertension, %	82.1	73.5	0.38
T2DM, %	46.2	44.1	0.86
Dyslipidemia, %	78.1	50	0.001
Family history of CAD, %	25.6	38.2	0.25
Current smoking, %	46.2	64.7	0.11
**Prior medication use**			
Aspirin, %	51.3	29.4	0.06
P2Y12 inhibitor, %	23.1	11.8	0.21
Statin, %	71.8	41.2	0.008
RASb, %	66.7	58.8	0.49
Beta blocker, %	56.4	38.2	0.12
**Laboratory results**			
Total cholesterol, mg/dL	211 ± 54	206 ± 42	0.65
LDL-C, mg/dL	127 ± 40	123 ± 33	0.64
HDL-C, mg/dL	35 ± 8	32 ± 7	0.10

BMI: body mass index, T2DM: type 2 diabetes mellitus, STEMI: ST elevation myocardial infarction, NSTEMI: non-ST elevation myocardial infarction, CAD: coronary artery disease, RASb: renin-angiotensin system blocker, LDL-C: low-density lipoprotein cholesterol, HDL-C: high-density lipoprotein cholesterol. Continuous variables are presented as mean ± standard deviation. Categorical variables are presented as percentages.

**Table 4:** Multivariable linear regression for determinants of NLRP3, Caspase-1 and IL-1β fold change at follow-up.

Predictor	NLRP3		Caspase−1		IL1β	
	B (95% CI)	*p*	B (95% CI)	*p*	B (95% CI)	*p*
Age	−0.14 (−0.77, 0.49)	0.58	−0.001 (−0.080.08)	0.97	−0.13 (−0.30, 0.03)	0.07
Male Sex	−12.75 (−49.58, 24.08)	0.39	3.21 (−0.89, 7.31)	0.11	−4.02 (−10.00, 1.96)	0.10
STEMI (vs. NSTEMI)	1.55 (−18.74, 21.84)	0.84	3.50 (1.00, 6.00)	0.012	1.09 (−2.12, 4.30)	0.28
LVEF	−1.45 (−3.23, 0.34)	0.09	0.06 (−0.07, 0.20)	0.32	−0.31 (−0.48, −0.15)	0.01
Hypertension	−41.62 (−90.28, 7.04)	0.08	2.79 (−1.52, 7.09)	0.17	−0.37 (−7.48, 6.73)	0.84
T2DM	−4.73 (−22.69, 13.23)	0.51	−1.35 (−3.96, 1.26)	0.27	−4.81 (−8.17, −1.46)	0.02
Dyslipidemia	−26.33 (−51.53, −1.12)	0.04	2.60 (0.96, 6.16)	0.13	−6.84 (−13.45, −0.23)	0.047
Smoking	10.45 (−19.97, 40.88)	0.39	0.76 (−3.54, 2.01)	0.54	−1.19 (−4.08, 1.69)	0.22
Peak TnI	−0.001 (−0.01, 0.01)	0.27	0.000 (−0.001, 0.000)	0.11	0.000 (−0.001, 0.001)	0.37
Total cholesterol	0.11 (−0.33, 0.54)	0.54	−0.03 (−0.08, 0.01)	0.14	−0.05 (−0.10, 0.006)	0.06
LDL-C	−0.13 (−0.61, 0.36)	0.51	0.08 (0.02, 0.14)	0.01	0.33 (0.08, 0.22)	0.01
HDL-C	1.18 (−0.48, 2.84)	0.12	−0.07 (−0.22, 0.07)	0.27	0.006 (−0.07, 0.08)	0.03
hsCRP	0.54 (0.02, 1.06)	0.045	0.02 (−0.03, 0.06)	0.78	0.006 (−0.07, 0.08)	0.75
BMI	−1.59 (−4.54, 1.37)	0.21	0.20 (−0.03, 0.42)	0.10	−0.006 (−0.34, 0.32)	0.94
Aspirin	27.17 (1.90, 52.44)	0.04	−2.30 (−4.85, 0.25)	0.07	−4.57 (−8.19, −0.94)	0.03
P2Y12 inhibitor	15.79 (−7.95, 39.54)	0.14	−2.89 (−5.76, −0.03)	0.048	−4.34 (−7.51, −1.17)	0.03
RASb	19.97 (−18.17, 58.10)	0.22	0.86 (−1.78, 3.50)	0.47	2.53 (−3.11, 8.16)	0.19
Statin	22.34 (−2.99, 47.66)	0.07	1.11 (−2.07, 4.29)	0.44	8.08 (1.67, 14.49)	0.03
Beta blocker	−22.46 (−42.63, −2.28)	0.04	−0.96 (−3.15, 1.23)	0.34	−0.10 (−2.83, 2.62)	0.89

LVEF: left ventricular ejection fraction, T2DM: type 2 diabetes mellitus, TnI: troponin I, LDL-C: low-density lipoprotein cholesterol, HDL-C: high-density lipoprotein cholesterol, hsCRP: high sensitivity C reactive protein, BMI: body mass index, RASb: renin-angiotensin system blocker.

## Data Availability

The datasets supporting the findings of this research are available from the corresponding author upon reasonable request.

## Supplementary Materials

The following supporting information can be downloaded at: https://www.mdpi.com/article/10.3390/life16010001/s1, Figure S1: Study flow diagram.
