# Supplementary figures and images for: Temporal Expression of NLRP3 Inflammasome Components in Patients with Acute Coronary Syndrome
Source: Life (Basel). 2025 Dec 19;16(1):1. doi: 10.3390/life16010001 (PMC12842691; doi:10.3390/life16010001)

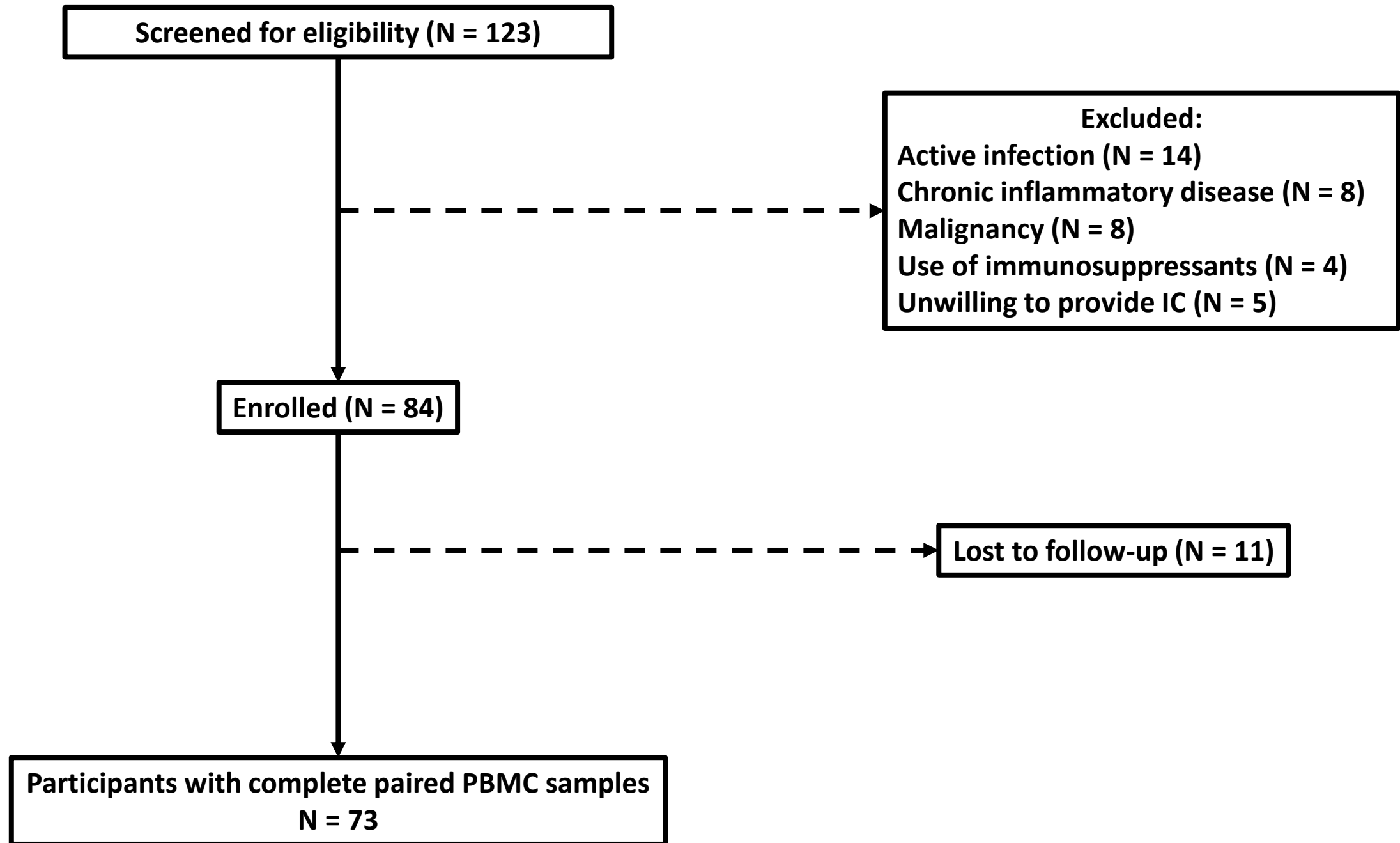

Supplement: Supplementary file 1 [file life-16-00001-s001.zip › life-4032989-supplementary.pdf]
